# Development and biomechanical analysis of an axially controlled compression spinal rod for lumbar spondylolysis

**DOI:** 10.1097/MD.0000000000038520

**Published:** 2024-06-07

**Authors:** Jingyuan Li, Zhifang Tang, Fanzhe Feng, Jinlong Liang, Nengqi Shao, Yulei Wang, Zhijun Cai, Hui Tang, Tianhua Zhou, Yongqing Xu, Yi Cui

**Affiliations:** a Clinical Medical College of Dali University, Dali, China; b Department of Orthopaedic Surgery, 920th Hospital of Joint Logistics Support Force, Kunming, China.

**Keywords:** biomechanics, lumbar spondylolysis, spinal rod

## Abstract

**Background:**

To elucidate the differences in mechanical performance between a novel axially controlled compression spinal rod (ACCSR) for lumbar spondylolysis (LS) and the common spinal rod (CSR).

**Methods:**

A total of 36 ACCSRs and 36 CSRs from the same batch were used in this study, each with a diameter of 6.0 mm. Biomechanical tests were carried out on spinal rods for the ACCSR group and on pedicle screw-rod internal fixation systems for the CSR group. The spinal rod tests were conducted following the guidelines outlined in the American Society for Testing and Materials (ASTM) F 2193, while the pedicle screw-rod internal fixation system tests adhered to ASTM F 1798-97 standards.

**Results:**

The stiffness of ACCSR and CSR was 1559.15 ± 50.15 and 3788.86 ± 156.45 N/mm (*P* < .001). ACCSR’s yield load was 1345.73 (1297.90–1359.97) N, whereas CSR’s was 4046.83 (3805.8–4072.53) N (*P* = .002). ACCSR’s load in the 2.5 millionth cycle of the fatigue four-point bending test was 320 N. The axial gripping capacity of ACCSR and CSR was 1632.53 ± 165.64 and 1273.62 ± 205.63 N (*P* = .004). ACCSR’s torsional gripping capacity was 3.45 (3.23–3.47) Nm, while CSR’s was 3.27 (3.07–3.59) Nm (*P* = .654). The stiffness of the pedicle screws of the ACCSR and CSR group was 783.83 (775.67–798.94) and 773.14 (758.70–783.62) N/mm (*P* = .085). The yield loads on the pedicle screws of the ACCSR and CSR group was 1345.73 (1297.90–1359.97) and 4046.83 (3805.8–4072.53) N (*P* = .099).

**Conclusion:**

Although ACCSR exhibited lower yield load, stiffness, and fatigue resistance compared to CSR, it demonstrated significantly higher axial gripping capacity and met the stress requirement of the human isthmus. Consequently, ACCSR presents a promising alternative to CSR for LS remediation.

## 1. Introduction

Lumbar spondylolysis (LS) is a common orthopedic disease characterized by discontinuous bone defects in the unilateral or bilateral isthmus of the lumbar spine, often resulting in lumbar spondylolisthesis.^[1]^ Both congenital factors and acquired trauma can trigger LS, with its symptoms typically manifesting as intractable lumbago, although some patients remain asymptomatic.^[1]^ LS most frequently occurs at the L5 level, followed closely by L4.^[1]^ Notably, 80% of patients experiencing lumbago present with bilateral spondylolysis.^[2]^ Factors contributing to LS encompass genetic predisposition and acquired traumatic incidents; for example, Eskimos show a higher incidence rate of LS, highlighting the influence of genetic factors. Acquired traumatic factors include stress fractures induced by intense and periodic physical activity, which is high among wrestlers and weightlifters.^[1]^

Currently, LS treatment options are categorized into conservative and surgical approaches.^[2]^ Surgical intervention is recommended if intractable low back pain persists after 6 to 12 months of conservative treatment.^[3]^ More than 10 surgical treatment options exist, including local bone grafting direct repair,^[4]^ single lag screw fixation (Buck method),^[5]^ Morscher screw internal fixation mechanism,^[6]^ transverse process-spinous process wire fixation (Scott method),^[7]^ pedicle screw-hook-rod fixation mechanism,^[8]^ pedicle screw-rod internal fixation mechanism,^[9]^ pedicle screw fixation mechanism,^[10]^ temporary short-segment fixation,^[11]^ and pedicle crew-hook-rod mechanism,^[12]^ all providing compression fixation. Among these, the pedicle screw-hook-rod internal fixation system is most commonly used,^[8]^ involving manual compression of the gap by the surgeon using handheld compression forceps. However, the variation in compression force applied by different surgeons may lead to the absence of tight alignment of the gap, leading to delayed union or nonunion.^[13]^ A wider gap will increase the risk of non-fusion after pars repair, posing a significant challenge in the field of spine surgery research.^[14]^ To realize precise gap compression, we designed and developed the axially controlled compression spinal rod (ACCSR) to achieve axial controllable compression, ensuring the gap tight alignment. This study aims to elucidate the mechanical performance between ACCSR and common spinal rod (CSR).^[15–17]^ We performed a series of biomechanical tests to confirm that this axially controlled compression spinal bar meets the necessary biomechanical strength for treating lumbar spondylolysis. This provides a strong basis for potential CSR replacement in the future.

## 2. Materials and methods

### 2.1. Development, composition, and parameters of ACCSR

**Figure 1.**
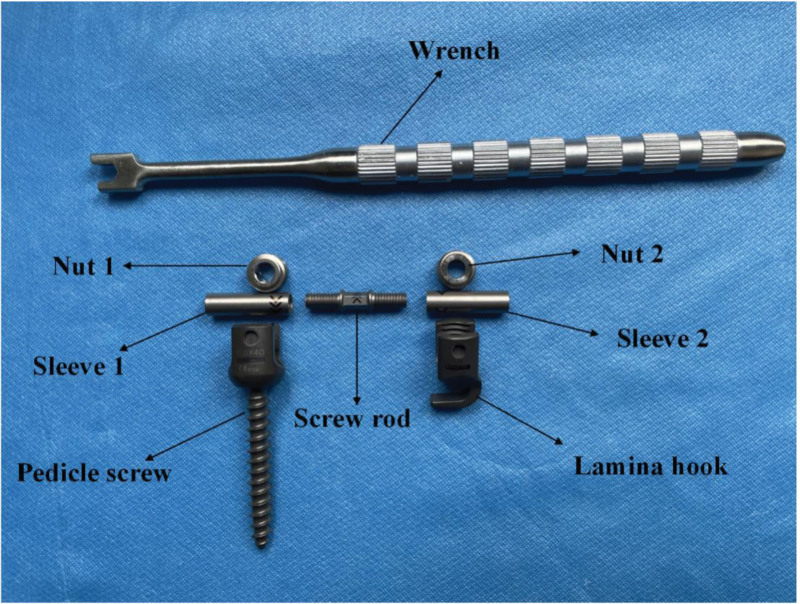
Schematic of the internal fixation system for lumbar spondylolysis.

The ACCSR utilized in this study was manufactured by WEGO ORTHO Materials Co., LTD. (Weihai, Shandong, China). It possesses a 6 mm diameter, featuring a central screw rod with reverse threads on both sides. The outer diameter measures 4.5 mm, while the inner diameter is 3.2 mm, and the total length is 8 mm. The length of the ACCSR for the experiment was selected in line with the American Society for Testing and Materials (ASTM) F2193 standards and ASTM F1798-97 standards. The screw rod can be nested by the sleeves on both ends, with the non-threaded section of the sleeves acting as a solid component (Fig. 1). The pressure mechanism involves rotating the screw using a wrench, causing the sleeves on both ends to move towards the screw, thus achieving an axial controllable pressure effect.

### 2.2. Experimental design and grouping

#### 2.2.1. Methodology

The mechanical testing was divided into 2 main parts. The first part focused on spinal rod testing, which included four-point bending tests and spinal rod fatigue tests, evaluating the mechanical performance of both ACCSR and CSR. The second part involved testing the mechanical interaction between spinal rods and pedicle screw-rods, including axial gripping capacity tests, torsional gripping capacity tests, and flexion/extension resistance tests. All biomechanical tests were carried out using an electromagnetic servo material testing machine (Instron ElectroPuls E10000 System; Instron Corporation, Massachusetts, USA) in accordance with ASTM standards.

#### 2.2.2. Trial-group assignments

All experimental SRs were produced from the same batch of medical titanium alloy, and tested at ambient room temperature with a custom-sized SR according to ASTM standards.

#### 2.2.3. Grouping for spinal rod test

The spinal rod tests were divided into 2 groups: the ACCSR group and the CSR group. A total of 15 ACCSR and 15 CSR samples were produced for these tests. Following the ASTM F2193 test standard, the spinal rods underwent four-point bending tests (using 60 mm × 6.0 mm spinal rods, 7 samples in each group) and spinal rod fatigue tests (using 60 mm × 6.0 mm spinal rods, with fatigue performance tested under 4 loads: 640 N, 400 N, 350 N, and 320 N, respectively. Two samples were tested under each load, totaling 8 samples in each group).

#### 2.2.4. Grouping for pedicle screw-rod internal fixation system test

The pedicle screw-rod internal fixation system tests were divided into ACCSR and CSR groups. A total of 21 ACCSR and 21 CSR samples were utilized for these experiments. The tests included axial gripping capacity tests (using 20 mm × 6.0 mm spinal rods, 7 samples in each group); torsional gripping capacity tests (using 60 mm × 6.0 mm spinal rods, 7 samples in each group), and flexion/extension resistance tests (using 80 mm × 6.0 mm spinal rods, 7 samples in each group). All tests used 6.0/40 mm pedicle screws, totaling 42 screws across the experiments.

### 2.3. Biomechanical test

#### 2.3.1. Biomechanical testing of spinal rods

##### 2.3.1.1. Four-point bending test

**Figure 2.**
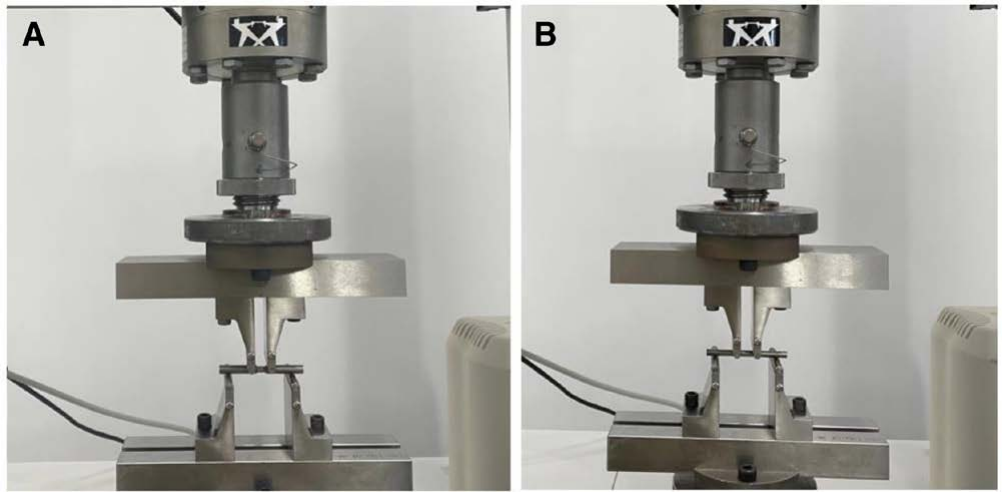
According to ASTM F2193 standards, the ACCSR (A) and CSR (B) groups underwent four-point bending and spinal rod fatigue tests. The stiffness and yield load of both groups were investigated. ACCSR = axially controlled compression spinal rod, ASTM = American Society for Testing and Materials, CSR = common spinal rod.

The four-point bending test was conducted in accordance with ASTM F2193 standards. Each spinal rod was evenly placed on the support rollers of the four-point bending fixture. The inner rollers were set at a span of 15 mm, with an additional 15 mm span between the inner rollers and the outer rollers on both sides (Fig. 2A and B). Each rod was placed on a four-point bent jig of the testing machine and subjected to axial pressure at a rate of 5 mm/min until failure occurred. The test was terminated upon the appearance of a breaking pattern. Failure was defined as any permanent deformation due to fracture or plastic deformation rendering the spinal rod ineffective or incapable of resisting the load. This included a reduction in compressive force, bending, or rupture. Load and displacement data were recorded at a sampling rate of 50 Hz throughout the test. Yield load and stiffness were calculated based on the experimental data.^[18]^

##### 2.3.1.2. Spinal rod fatigue test

The fatigue test, following ASTM F2193 standards, comprised 2.5 million cycles. The initial maximum fatigue moment level was set at 25% (640 N) of the ultimate bending moment value determined through the static test method recommended for fatigue research. Each rod was fixed on the fatigue testing machine, and axial pressure was applied at a frequency of 5 Hz at a constant speed. If failure or fracture of the internal fixation systems occurred before completing 2.5 million cycles, it was considered a test failure. The compression load was reduced until passing the 2.5 million loading cycle test.^[19,20]^ The difference between the maximum bending moment leading to the failure of the test sample and the maximum terminating bending moment should be less than 10% of the ultimate bending moment of the sample. The loading period during each fatigue test failure and the loading compression load upon success were recorded and compared with the maximum force of the unilateral lumbar isthmus in normal adults, which does not exceed 198.72 N.^[21]^

#### 2.3.2. Biomechanical test of pedicle screw-rod internal fixation system

##### 2.3.2.1. Axial gripping capacity test

**Figure 3.**
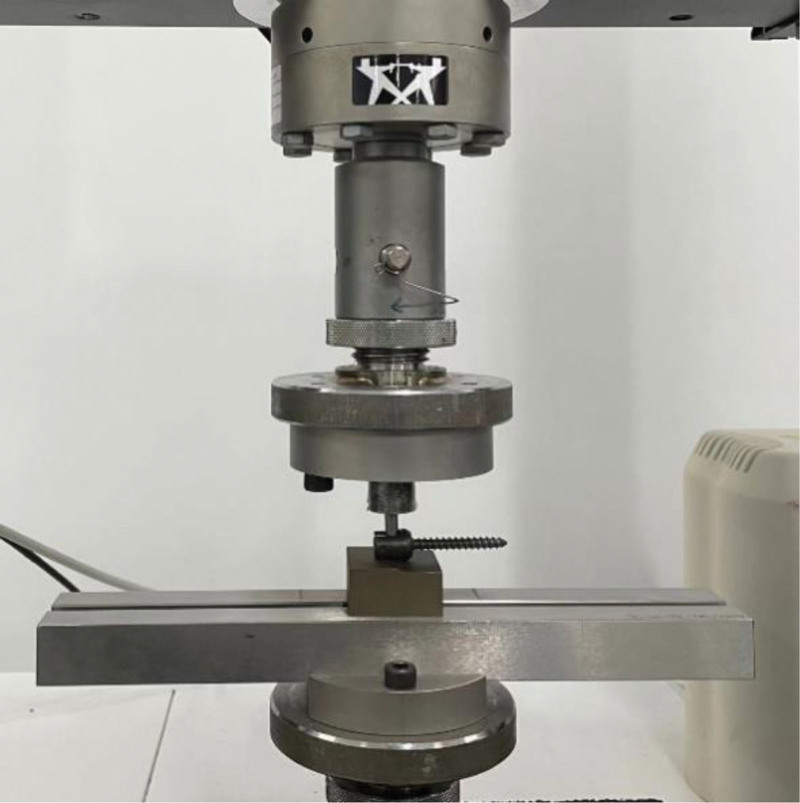
According to ASTM F1798-97 standards, this fixture was used for the axial gripping capacity test. The axial gripping capacity of the nail-rod structure was determined by applying an axial load to the pedicle screw-rod. ASTM = American Society for Testing and Materials.

In both ACCSR and CSR groups, a single pedicle screw was connected to the spinal rod using a nut, following ASTM F1798-97 standards. The nut was tightened with a torque of 12 Nm as per industry standards. The pedicle screw was affixed to the test bench (Fig. 3). An instrument applied a constant axial load to one end of the spinal rod at a constant speed of 2 mm/min. The displacement greater than 3 mm was measured, and the maximum thrust load was recorded.^[22,23]^ The axial gripping capacity of both groups (maximum thrust load within 1.5 mm of initial displacement) was calculated.

##### 2.3.2.2. Axial torsion test

**Figure 4.**
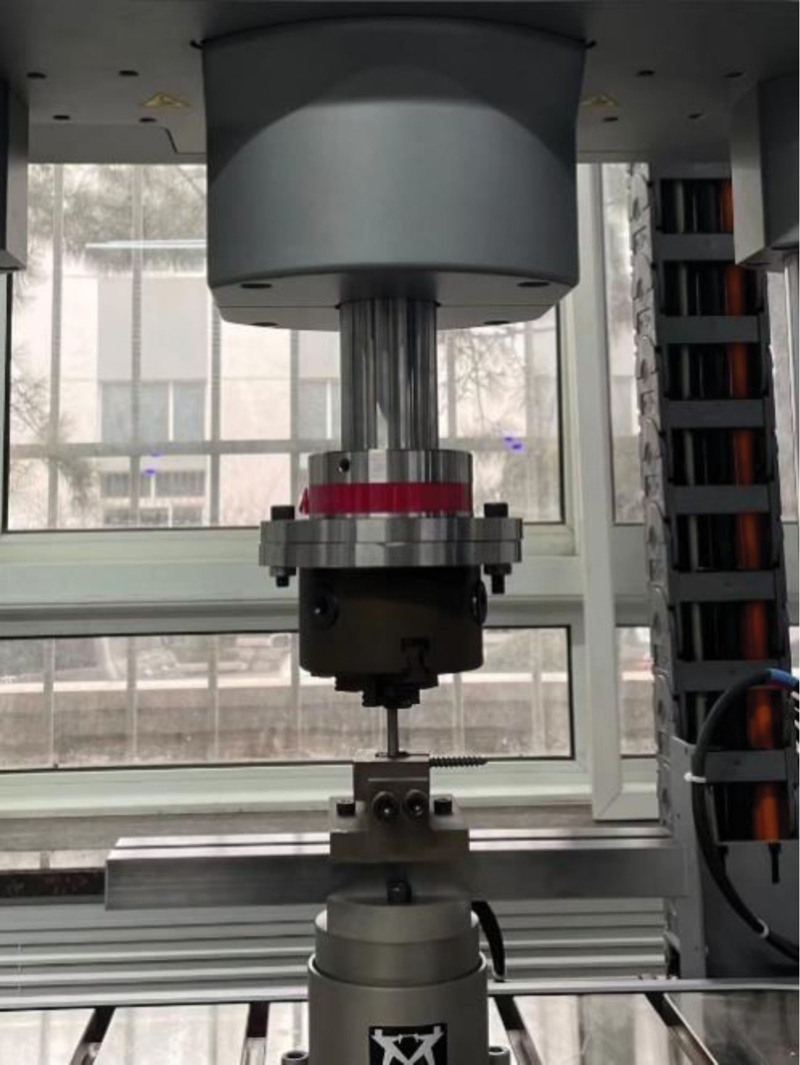
The fixture for the torsional gripping capacity test, as per ASTM F1798-97 standards. Torque was applied to the rod to assess the torsional gripping capacity of the pedicle screw-rod structure. ASTM = American Society for Testing and Materials.

In accordance with the ASTM F1798-97 test standard, a single pedicle screw was connected to the spinal rod in both the ACCSR and CSR groups using a nut. Following industry protocols, the nut was screwed into the pedicle screw at a torque of 12 Nm and securely locked, forming the basic fixation unit. The pedicle screw-rod structure was then fixed axially onto the test table. Torsion was applied at a rate of 20°/min. Measurements of angular displacement and torque were recorded (Fig. 4). The torsional gripping capacity, indicating the maximum torque within 10° before rotation, was calculated based on the collected data.^[22]^

##### 2.3.2.3. Flexion/extension resistance tests

**Figure 5.**
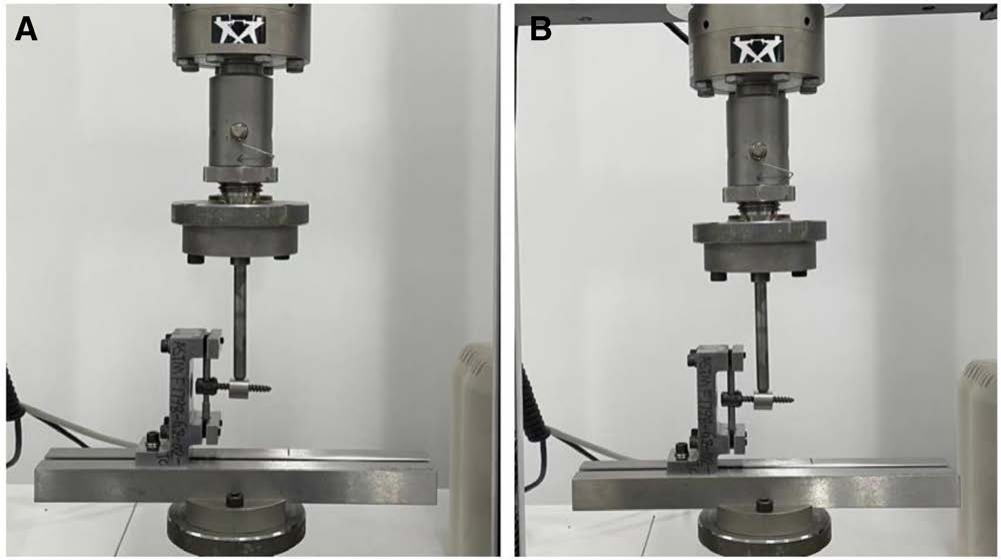
This fixture, compliant with ASTM F1798-97 test standards, the ACCSR (A) and CSR (B) groups underwent flexion/extension resistance tests. Axial load was applied to the pedicle screw to assess stiffness and yield load in the pedicle screw in the pedicle screw-rod configuration. ACCSR = axially controlled compression spinal rod, ASTM = American Society for Testing and Materials, CSR = common spinal rod.

Following the ASTM F1798-97 test standard, the nuts were screwed into the pedicle screw and secured at a torque of 12 Nm, forming the basic fixation unit along with the spinal rod. The pedicle screw-rod structure was axially fixed on the test table, ensuring the nail tail was horizontal. A 1 cm long ring was placed in the middle of the nail tail, serving as the axial stress loading point (Fig.5A and B). Axial stress was applied at a rate of 2 mm/min at a distance of 25 mm from the calibration point. The axial pressure was applied until the pedicle screw failed. Stiffness and yield load of the pedicle screw were measured during this process.^[24,25]^

### 2.4. Statistical methods

Data processing and statistical analyses were conducted using SPSS 26.0 (IBM, Armonk, NY). Measurement data conforming to a normal distribution were presented as mean ± standard deviation (x̄ ± s), with a comparison between the 2 groups performed using the 2 independent sample *t* tests. For non-normally distributed measurement data, median and quartile values were used. Intergroup comparisons were conducted using the rank sum test, with statistical significance set at *P* < .05.

## 3. Results

All data were processed using Origin software.

### 3.1. Biomechanical test of spinal rod

#### 3.1.1. Results of the four-point bending test

The stiffness and yield load for the ACCSR group were 1559.15 ± 50.15 N/mm and 1345.73 (1297.9–1359.97) N, respectively. In comparison, the CSR group exhibited a stiffness of 3788.86 ± 156.45 N/mm and a yield load of 4046.83 (3805.84072.53) N. Significant differences were observed between the ACCSR and CSR groups (Table 1, *P* < .05).

**Table 1:** Results of four-point bending test, axial gripping capacity test, torsional gripping capacity test, and flexion/extension resistance test for ACCSR group and CSR group.

	n	ACCSR	CSR	Z/*t*	*P* value
Stiffness of four-point bending test, N/mm	7	1559.15 ± 50.15	3788.86 ± 156.45	−35.907	<.001
Yield load of four-point bending test, N	7	1345.73 (1297.90–1359.97)	4046.83 (3805.8–4072.53)	−3.130	.002
Axial gripping capacity, N	7	1632.53 ± 165.64	1273.62 ± 205.63	3.596	.004
Torsional gripping capacity, Nm	7	3.45 (3.23–3.47)	3.27 (3.07–3.59)	−0.448	.654
Stiffness of flexion/extension resistance test, N/mm	7	783.83 (775.67–798.94)	773.14 (758.70–783.62)	−1.725	.085
Yield load of flexion/extension resistance test, N	7	651.3 ± 14.22	618.88 ± 43.1	1.890	.099

ACCSR = axially controlled compression spinal rod, CSR = common spinal rod.

#### 3.1.2. Results of spinal rod fatigue test

**Figure 6.**
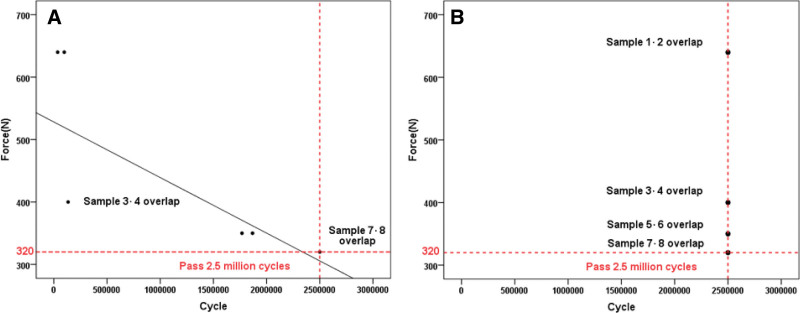
Plots depicting maximum compression load-cycles and corresponding fatigue limits for single rod fatigue tests in the ACCSR group (A) and CSR group (B), surpassing 2.5 million cycle loads. ACCSR = axially controlled compression spinal rod, CSR = common spinal rod.

During the spinal rod fatigue test, both groups were subjected to loads of 640 N, 400 N, 350 N, and 320 N. The test results indicated that the CSR group endured over 2.5 million cycles under various loads (Fig. 6B). The ACCSR group completed 2.5 million fatigue tests under a 320 N load, exceeding 198.72 N (Fig. 6A). This demonstrates that the device can fulfill the stress requirements for spondylolysis.

### 3.2. Biomechanical test of pedicle screw-rod internal fixation system

#### 3.2.1. Results of axial gripping capacity test

The average axial gripping capacity (maximum holding force within 1.5 mm of slip) was 1273.62 ± 205.63 N for the CSR group and 1632.53 ± 165.64 N for the ACCSR group. Significant higher gripping capacity was observed in the ACCSR group compared to the CSR group (Table 1, *P* < .05).

#### 3.2.2. Results of torsional gripping capacity test

The torsional gripping capacity (maximum torque within 10° before rotation) for the CSR group and ACCSR group were 3.27 (3.07–3.59) and 3.45(3.23–3.47) Nm, respectively. No statistically significant difference was found between the 2 groups (Table 1, *P* = .655 > .05).

#### 3.2.3. Results of flexion/extension resistance test

The stiffness for the CSR group and ACCSR group were 773.14 (758.70–783.62) and 783.83(775.67–798.94) N/mm, respectively. Additionally, the yield loads were 618.88 ± 43.1 and 651.3 ± 14.22 N for the CSR group and ACCSR group, respectively. No significant difference was noted between the groups (Table 1, *P* > .05).

## 4. Discussion

The primary objective of advanced endo-plants is to expedite the reestablishment of physiological function. Simple LS repair preserves segmental physiological function, significantly improving patients’ quality of life. Despite the availability of various surgical methods for LS, a challenge remains: the precise compression of the gap, which is crucial for successful healing. Therefore, enhancing the healing rate and efficiency of LS has become a new development direction in LS surgical repair. The novel implant designed in this study represents a significant advancement in LS treatment.

### 4.1. Design concept and innovation of ACCSR

Previous studies both domestically and internationally^[12,26,27]^ have reported fusion rates in patients with lumbar spondylolysis treated via various methods such as the Buck group, Scott group, Morscher group, and pedicle screws (including pedicle screw-rod internal fixation mechanisms, screw-hook-rod internal fixation mechanisms of the pedicle, and modified Scott techniques). These methods exhibited fusion rates of 83.53%, 81.57%, 77.72%, and 90.21%, respectively. Notably, the pedicle screw group demonstrated the highest fusion rate, while the Morscher screw group displayed the lowest. Complication rates were 13.41%, 22.35%, 27.42%, and 12.8%, for the Buck, Scott, Morscher, and pedicle screw groups, respectively. The Buck group was more susceptible to nerve root stimulation, Scott repairs carried risks of wire and transverse process fractures, and the Morscher screw exhibited a higher incidence of prosthesis loosening. The pedicle screw-rod internal fixation mechanism, applies pressure through the spinous process, which may lead to fractures under excessive stress. Interestingly, the pedicle screw group demonstrated the lowest incidence of complications. None of these surgical procedures can achieve controlled axial compression at the isthmic end, prompting the development of ACCSR. Li et al^[28]^ demonstrated the superior stability of the pedicle screw-hook-rod internal fixation system, leading us to continue its use in our research and development efforts. Our innovative approach, ACCSR, adheres to the AO fracture healing standard.^[13]^ ACCSR offers several advantages: ACCSR, aided by intraoperative C-arm fluoroscopy and direct visual observation, allows controllable compression facilitating anatomical reduction of the gap during surgery; By enabling continuous compression, ACCSR ensures tight closure of the fracture ends, securing initial stability and promoting bone graft fusion in later stages; ACCSR requires exposure of only the spondylolysis, the lower lamina edge, and the superior facet joint of the isthmic vertebral body, eliminating the need to expose the transverse process and the inferior facet joint; and Post-surgery, patients can wear a brace to get out of bed on the first day after surgery, and engage in early mobilization, a strategy that aligns with similar successful approaches reported in other studies, such as Allende et al^[29]^ use of double tension band fixation for distal humeral fractures. The mechanism involves the use of tension band compression to bring together the fractured ends, thereby achieving controllable compression on the fracture sites to prevent elbow joint deformities, and demonstrates that controlled compression facilitates the anatomical reduction of fracture ends with significant clinical efficacy.

In this study, the biomechanical performances of ACCSR and its internal fixation systems were investigated using internal fixation strength tests, fatigue tests, and intraoperative observations. Yamada et al^[20]^ conducted a comprehensive assessment of titanium alloy SR and cobalt chromium molybdenum SR through four-point bending tests and spinal rod fatigue tests. They determined the yield load and stiffness value for each group, enabling a detailed evaluation of the rod’s fatigue performance under specific loads, thereby assessing its durability. Massey et al^[30]^ performed static compression and rotational experiments on 5.5-mm nitinol rods and 5.5-mm titanium rods to compare their construct stiffness and torsional toughness. Kluck et al^[22]^ evaluated the clamping ability of pedicle screws from 5 suppliers using shaft push tests and torsional gripping capacity tests. The mechanical performance of internal fixation systems was further assessed through flexion/extension resistance tests.^[25]^ Schwab et al^[31]^ reported that after posterior placement of the pedicle screw, the load on the spine is inevitably transferred to the internal fixation system, resulting in stress. Therefore, the configuration of the internal fixation systems is particularly important to prevent implant failure, such as dislocation between the SR and the pedicle screw, or loosening and breakage of the internal fixation. To evaluate the biomechanical properties of the pedicle screw-rod internal fixation system, axial gripping capacity tests, torsional gripping capacity tests, and flexion/extension resistance tests were evaluated.

### 4.2. Biomechanical performances of spinal rod

The results of the four-point bending test indicate that ACCSR exhibits a weaker yield load compared to CSR. This disparity arises due to stress concentration caused by the thread structure on both sides of the screw. However, this structure can be controlled to achieve stability within the gap. Additionally, progressive pressure control simplifies and facilitates compression at the gap. In evaluating the fatigue performance of a single bar, it was observed that ACCSR’s fatigue resistance falls below that of CSR. However, Adams^[32]^ found that the maximum load of the lumbar spine of normal adults who could bear lifting heavy objects was 700 N. Further analysis revealed that 80% of the stress was borne by the anterior column of the lumbar spine, while the middle and posterior columns bore 20% of the stress. Hence, the maximum load of the middle column and posterior column was calculated to be 140 N.^[33]^ Notably, the maximum stress exerted on unilateral lumbar spine spondylolysis does not exceed 198.72 N.^[21]^ ACCSR, having successfully undergone 2.5 million fatigue tests with a load of 320 N, demonstrates its superior strength, that is 320 N > 198.72 N > 140 N. Research has indicated that^[34]^ individuals undergo an average of 4400 spinal movements daily, suggesting a durability of at least one and a half years. A study by CAI Zhijun et al^[35]^ revealed that patients treated with pedicle screw-hook-rod internal fixation systems for LS exhibited promising outcomes. Approximately 95% of these patients achieved isthmic bone healing within an average of 9.7 months post-surgery, underscoring the system’s robust fatigue resistance that aligns with clinical demands.

### 4.3. Biomechanical performance of pedicle screw-rod internal fixation systems

The torsional gripping capacity test results indicated no significant difference between the ACCSR group and the CSR group (*P* > .05). However, ACCSR exhibited significantly higher axial gripping capacity than CSR. This suggests that the axial locking ability of ACCSR with the pedicle screw was superior to that of CSR, making it a safer choice for implantation in the human body. ACCSR is less prone to slipping between pedicle screws. The flexion/extension resistance test revealed no significant difference in the yield load and stiffness of the pedicle screws between the 2 internal fixation systems. This indicates that the mechanical performance of the pedicle screws remains unchanged after ACCSR application, making them resistant to breakage.

### 4.4. Intraoperative procedure of the pedicle screw-hook-rod internal fixation system with ACCSR

In this study, ACCSR was divided into 3 parts: the sleeve on both sides and the screw rod in the middle. The procedure began with the measurement of the required SR length using the die rod. The appropriate ACCSR, matching the measured length, was selected. The gap width of the spondylolysis was measured with a vernier caliper. The threads on both sides of the screw were adjusted in advance to align with the gap width. They were then exposed for an additional turn, maintaining consistency in length. The tail of the polyaxial pedicle screw and the lamina hook were adjusted to ensure vertical placement of ACCSR at the broken end, and ACCSR was plying-up with pedicle screws and laminar hooks to achieve optimal fixation.^[36]^ Subsequently, the pedicle screw and the nuts on the lamina hook were secured onto the sleeves on both sides. Using a wrench, the screw rod was rotated to achieve precise coaxial compression. With the rotation of the wrench, the 2 ends of the gap gradually approached each other until complete closure. The screw rod was then turned one more time to bring the broken end closer, ensuring a tight closure of the gap. The operation process of ACCSR is simple and provides convenience for the operator. It eliminates issues associated with manual compression using CSR pressure forceps, such as inaccurate compression force, incomplete gap closure, and inconvenient operation.

## 5. Conclusions

Although this trial validated the biomechanical strength of ACCSR for stabilizing LS, there are some limitations that should be acknowledged. Primarily, the biomechanical performance of the internal fixation systems was exclusively tested in vitro. Future research will use the finite element simulations of ACCSR to analyze its biomechanical properties. This will provide a comprehensive understanding of ACCSR’s efficacy in pressurizing the fracture end of spondylolysis and enhancing the healing rate. Our goal is to address the challenge of nonunion and delayed union of LS.

## Acknowledgments

Thanks to all the authors of this article for their outstanding contributions to this article.

## Author contributions

**Conceptualization:** Yi Cui.

**Data curation:** Nengqi Shao, Yulei Wang.

**Formal analysis:** Fanzhe Feng, Jinlong Liang.

**Resources:** Zhijun Cai, Hui Tang, Tianhua Zhou.

**Supervision:** Yongqing Xu.

**Writing – original draft:** Jingyuan Li, Zhifang Tang.
